# Lithium in Drinking Water as a Public Policy for Suicide Prevention: Relevance and Considerations

**DOI:** 10.3389/fpubh.2022.805774

**Published:** 2022-02-17

**Authors:** Pablo Araya, Camila Martínez, Jorge Barros

**Affiliations:** ^1^Department of Escuela de Medicina PUC School of Medicine, Facultad de Medicina, Pontificia Universidad Católica de Chile, Santiago, Chile; ^2^Departamento de Psiquiatría, Facultad de Medicina, Pontificia Universidad Católica de Chile, Santiago, Chile

**Keywords:** lithium, suicide, primary prevention, ethics, public policy, water supply

## Abstract

Although suicide is considered a major preventable cause of mortality worldwide, we do not have effective strategies to prevent it. Lithium has been consistently associated with lowering risk of suicide. This effect could occur at very low concentrations, such as trace doses of lithium in tap water. Several ecological studies and recent meta-analysis have suggested an inverse association between lithium in water and suicide in the general population, with a lack of knowledge of clinically significant side effects. This paper is aimed as a proposal to discuss the addition of lithium to drinking water to decrease the suicide rate. For this, we review the evidence available, use previous experiences, such as water fluoridation to prevent dental caries, and discuss the complexity involved in such a public policy. Considering the limited data available and the controversies contained in this proposal, we suggest that a consensus on lithium concentration in water is needed, where the suicide rates start to reduce, as happened with water fluoridation. This measure will require to develop community-controlled trials with strict monitoring of any side effects, where democratic procedures would constitute one of the most appropriate ways to validate its implementation according to the reality of each community.

## Introduction

Suicide is considered a major preventable cause of mortality worldwide, accounting for approximately more than 8,00,000 deaths per year (1, 2), and is among the top ten causes of age-standardized years of life lost in many regions across the world (2). Although there has been a substantial decrease in suicide mortality in recent decades, global and regional analysis can mask country-specific changes in suicides rates (1). Only 3% of 118 countries would achieve the goal of reducing suicide mortality from the Sustainable Development Goals by 2030 with the current trends (3).

Suicide is a complex and multifactorial phenomenon (1, 2). While suicide is not a mental illness itself, one of its main risk factors is having one (4). The incidence of suicide attempts during a major depressive episode or a mixed maniac episode can be 20–40 times higher compared to a euthymic mood (5). Other risk factors for developing suicidal behavior include previous suicide attempt, lower educational and income levels, single marital status, unemployment, parental psychopathology, and childhood adversity (4).

Most suicides occur in low- and middle-income countries where resources and services, if they do exist, are often scarce and limited (1). These unexpected deaths, that predominantly occur in young and middle-aged adults, result in a huge economic, social, and psychological burden for individuals, families, communities, and countries (1). In high developed countries, these population resulted in a loss of 406,730 years of life at a cost of $5.53 billion in lost economic income with the average cost of suicide estimated at $802,939 (6).

Despite advances in research and knowledge about suicide, many times health systems and services do not provide enough tools for timely and effective help (7). Along with individual strategies, a series of population measures have been proposed to prevent suicide, such as restricting access to pesticides and firearms, generating responsible information from the media, and designing policies aimed at reducing alcohol consumption (1).

Recent population studies suggests that the lithium found in drinking water could reduce the risk of suicide and possibly contribute as a mood stabilizer (8–10). Although at the individual level lithium has been described as one of the main drugs associated with a lower risk of suicide (11), its use at the population level implies a series of considerations.

This paper is aimed as a proposal to discuss the addition of lithium to drinking water as a public policy for suicide prevention, appealing to mental health professionals who fulfill advocacy roles to evaluate its relevance and the several factors and controversies implicated.

## Indications, Dosage, and Adverse Effects of Lithium

Lithium is used for the control and prevention of maniac and depressive episodes, acting as a mood stabilizer in people with bipolar disorder, as well as an adjunctive therapy in patients with major depressive disorder and schizoaffective disorder (12). It has been described how lithium is associated with lower risk of suicide and mortality from any cause in people with mood disorders (9, 11). Additionally, lithium would have a neuroprotective effect (13, 14) and a role as a reducer of aggressiveness and impulsivity (15). These mechanisms, however, has not been fully characterized (16).

Lithium should be dosed to clinical efficacy (17). In adults, it is usually dosed to obtain a serum therapeutic range of 0.6–1.0 mmol/L for chronic treatment of mood disorders (18) through 600 to 1,200 mg of lithium carbonate daily, with 300 mg of lithium carbonate containing 8 mmol of lithium approximately. It has been reported that lithium can improve and stabilize mood at doses up to 0.005 mmol (400 μg) per day in patients with a history of substance use disorder (19), suggesting that this could have an effect at doses of traces. Recently, a large cases series has described similar results with 4 mmol of lithium per day in a similar group of patients (20).

Regarding the dose associated with reducing suicide risk, the evidence is heterogeneous and limited by the relatively low number of events (death by suicide) within the group to be studied (9, 11). In a systematic review by Cipriani et al. (11), doses are reported to reach lithium levels in the blood between 0.3 and 1.6 mmol/L. Del Matto et al. (9) report doses for reaching levels between 0.4 and 1.0 mmol/L in prospective studies and 0.5–0.7 mmol/L in retrospective studies.

The adverse effects of lithium are dose dependent (12). Common adverse effects at therapeutic levels include tremor, hypothyroidism, weight gain, nausea, and vomiting (12, 21). Serious renal adverse effects are rare and associated with chronic lithium use for decades in therapeutic doses, where there are nonspecific interstitial fibrosis and gradual decrease in glomerular filtration rate (22). Lithium toxicity usually occurs with plasma levels >1.5 mmol/L, so it is necessary to monitor plasma levels in patients with pharmacological doses (12).

## Natural Experiences and Ecological Studies of Lithium as a Suicide Protective Agent

Schrauzer and Shrestha, in 1990, were one of the first to report that the incidence of suicide, rape and violence was significantly lower in counties whose lithium levels in drinking water ranged from 70 to 160 μg/L (0.0101–0.0244 mmol/L) in contrast to those with 0–12 μg/L (0 to 0.0017 mmol/L), over a 10-year period (23).

One of the highest concentrations of natural lithium salt deposits in the world are in the northern regions of Chile and Argentina (24, 25), accounting for more than the 50% of its global reserves (26), as well as one of the highest concentrations of lithium in surface waters, reaching concentrations between 1000 and 3000 μg/L (0.1441–0.4323 mmol/L) in Chile (24) and 10 to 1,000 μg/L (0.0014–0.1441 mmol/L) in Argentina (27). In Chile, König et al. (28) reported that the Atacama Region, the one that concentrates the highest amount of lithium in the country, has a significantly lower suicide rate compared to other regions after adjusting for socioeconomic variables, with 9.99 deaths per 100,000 inhabitants vs. 12.5 per 100,000, respectively. In Argentina, in contrast, López Steinmetz et al. (29) found an inverse association to what was initially expected, where higher suicide rates were reported in those localities with the highest concentration of lithium in drinking water, with concentrations between 70 μg/L to 1,650 μg/L (0.0101–0.2378 mmol/L) and a mortality rate due to suicide between 19.12 suicides per 100,000 inhabitants and 30.22 suicides per 100,000 inhabitants respectively.

In the systematic review and meta-analysis carried out by Barjasteh-Askari et al. (8), a total of 13 studies were analyzed, reporting a significant relationship between the lithium concentration in drinking water and reduced suicide mortality in men and general population. Mean lithium levels were between 3.8 and 123 μg/L (0.0005–0.0177 mmol/L) and ranged from 0.1 to 43 μg/L (0.00001–0.0062 mmol/L) to 0–160 μg/L (0–0.0231 mmol/L). The studies that found no association ranged from 0 to 12.9 μg/L (0–0.0019 mmol/L) to 0–191 (0–0.0275 mmol/L). The suicide mortality data considered periods between 1 and 11 years. Of the total ecological studies, four were conducted in Japan, three in the United States, and one in each of the following countries: Austria, England, Greece, Italy, Lithuania, Denmark, and Portugal.

Del Matto et al. (9) carried out a systematic review with a total of 16 ecological studies reported. The authors concluded that 11 of them found that higher levels of lithium in drinking water were associated with a lower suicide rate. Lithium levels were heterogeneous between the studies, with mean levels between 3.8 and 46.3 μg/L (0.0005–0.0067), and ranges from 0.1 to 43 μg/L (0.00001–0.0062 mmol/L) to 3 to 539 μg/L (0.0004–0.0778 mmol/L). Among the studies that found no association, the lithium dose ranged from 0.6 to 30.7 μg/L (0.0001–0.0044 mmol/L) to 0–191 μg/L (0–0.0275 mmol/L). The suicide mortality data considered periods between 3 months to 6 years. Of the total ecological studies, five were conducted in Japan, three in Austria, two in the United States, and one in each of the following countries: England, Greece, Italy, Lithuania, Denmark, and Portugal.

In the systematic review and meta-analysis carried out by Memon et al. (10), nine ecological studies were considered, where a significant inverse association was found between lithium levels in public drinking water and the suicide mortality rate in women and the total population. The mean levels of lithium in the water were between 3.8 and 46.3 μg/L (0.0005–0.0067 mmol/L) and ranged from 0.1 to 43 μg/L (0.00001–0.0062 mmol/L) to 2.8 to 219 μg/L (0.0004–0.0316 mmol/L). Among the studies that found no association, the ranges were between from 0 to 12.9 μg/L (0–0.0019 mmol/L) to 0.11 to 60.8 μg/L (0.00002–0.0088 mmol/L). The suicide mortality data considered periods between 1 and 11 years, with a range between 7.53 and 27 suicides per 100,000 inhabitants by year. Of these nine studies, two were conducted in the United States, two in Japan, and one in each of the following countries: England, Austria, Greece, Italy, and Lithuania.

The characteristics of the ecological studies included in the reviews aforementioned are summarized in Table 1 (23, 30–47). Among these, Knudsen et al. (42) analyzed lithium exposure on an individual level calculated as a moving five-year time-weighted average over a 22-year period, reporting no significant association between lithium exposure and suicide rate. The authors concluded that there does not seem to be a protective effect of exposure to lithium on the incidence of suicide with levels below 30.7 μg/L (0.0044 mmol/L) in drinking water. Although this study may offer a more thorough methodological approach than the other ecological studies, it should be considered that the evidence that suggests a significant association with lower risk of suicide reports usually higher lithium levels, with up to 32.9 μg/L (0.0047 mmol/L) (47), 35.53 μg/L (0.0051 mmol/L) (44), 43 μg/L (0.0062 mmol/L) (43), 59 μg/L (0.0085 mmol/L) (31), 82.3 μg/L (0.0119 mmol/L) (33), 121 μg/L (0.0175 mmol/L) (37), 130 μg/L (0.0188 mmol/L) (40), 160 μg/L (0.0231 mmol/L) (23), and 219 μg/L (0.0316 mmol/L) (36). Conversely, studies with lower levels of lithium up to 12.9 μg/L (0.0019 mmol/L) (38) and 21 μg/L (0.0030 mmol/L) (32) did not find an association. Exceptions to this observation are the studies carried out by Dawson et al. (30), Hellbich et al. (39), Pompili et al. (41), and Oliveira et al. (46).

**Table 1:** Characteristics of the studies of lithium at trace doses as a suicide protective agent.

**Author, year**	**Review included in**	**Geographical area**	**Population data**	**Lithium levels, mean (range)**	**Lithium samples, *n*; dates and methods of collection; analysis method**	**Mean SR per 100,000/year, mean (range)**	**SMR of suicide, mean (range)**	**Results, statistical methods and covariates**
Dawson et al. (30)	Barjasteh-Askari et al. (8)	Texas, USA, 24 counties	All suicides 1968–1969 (total population n.r.)	29.37 μg/L (0–139 μg/L) [0.0042 mmol/L (0–0.0201 mmol/L)]	*n* = n.r.; date n.r., from publicly accessible water sources; optical spectrometry	9.75	n.r.	NA between [Li] and SMR Pearson correlation between Li and SR T: r = −0.235; *p* > 0.05 Covariates: n.r.
Schrauzer and Shrestha (23)	Barjasteh-Askari et al. (8)	Texas, USA, 27 counties	All suicides 1978–1987 (total population 10 068 000)	Group A: 123 μg/L (70–160 μg/L) [0.0177 mmol/L (0.0101–0.0231 mmol/L)] Group B: 35 μg/L (13–60 μg/L) [0.0050 mmol/L [0.0019–0.0086 mmol/L)] Group C: 5 μg/L (0–12 μg/L) [0.0007 mmol/L (0–0.0017 mmol/L)]	*n* = n.r.; date n.r., from municipal water supplies; method n.r.	13.13	n.r.	Less suicide in the higher [Li] group Student's *t*-test between groups, with Bonferroni correction for multiple comparisons T: IR_A_ = 8.7 ± 0.85; IR_B_ = 14.8 ± 2.9; IR_C_ = 14.2 ± 1.3; P A-B <0.005; P A-C <0.01; P B-C>0.05 Covariates: n.r.
Ohgami et al. (31)	Barjasteh-Askari et al. (8) and Del Matto et al. (9)	Oita, Japan, 18 municipalities	All suicides 2002–2006 (total population 1 206 174)	n.r. (0.7–59 μg/L) [n.r. (0.0001–0.0085 mmol/L)]	*n* = 18; date n.r., from tap water supplies; chromatography and mass spectroscopy	n.r.	105 (60–181)	Less suicide with higher [Li] in total population and males, but not in females PWLS regression of SMR on log Li T: β = −0.65; *p* < 0.004 M: β = −0.61; *p* < 0.008 F: β = −0.46: 0.055 < *p* < 0.06 Covariates: n.r. (psychosocial and economic factors were not taken into consideration)
Kabacs et al. (32)	Barjasteh-Askari et al. (8), Del Matto et al. (9) and Memon et al. (10)	East of England, 47 subdivisions	All suicides 2006–2008 (total population 5 700 000)	4.98 μg/L (<1–21 μg/L) [0.0007 mmol/L (<0.0001–0.0030 mmol/L)]	*n* = 47; during 2010, from publicly accessible water sources; mass spectrometry	n.r.	T: 98 (36–194) M: 95 (35–213) F: 108 (0–292)	NA between [Li] and SMR Pearson correlation between Li and SMR T: r = −0.03; *p* = 0.838;M: r = −0.054; *p* = 0.715; F: r = 0.042; *p* = 0.777 PWLS regression of SMR on log Li T: β = −0.062, s.e. = 0.145 M: β = −0.059, s.e. = 0.143 F: β = −0.036, s.e. = 0.147 Covariates: n.r.
Kapusta et al. (33)	Del Matto et al. (9) and Memon et al. (10)	Austria, 99 districts	All suicides 2005–2009 (total population 8 297 964)	11.3 μg/L (3.3–82.3 μg/L) [0.0016 mmol/L (0.005–0.0119 mmol/L)]	*n* = 6,460; 2005–2010, from local drinking water; ICP-OES	T: 16.5 M: 26.4 F: 7.00	T: 0.790 M: 0.821 F: 0.673	Less overall SR and SMR with higher [Li] PWLS regression of SMR on log Li Unadjusted analyses T: β = −0.22; *p* = 0.029; M: β = −0.18; *p* = 0.083; F: β = −0.21; *p* = 0.03 Adjusted analyses T: β = −0.243; *p* = 0.022; M: β = −0.19; *p* = 0.062; F: β = −0.22; *p* = 0.088 Covariates: Population density, income per capita, proportion of Roman Catholics, unemployment rates, density of GPs, psychotherapists and psychiatrist
Schopfer and Schrauzer (34)	Del Matto et al. (9)	Tokyo, Japan	n.r.	M: 0.0190 μg/g F: 0.0275 μg/g	*n* = 200; date n.r., from scalp hair; hair analysis (Samples taken at a clinic of preventive medicine on healthy individuals)	n.r.	n.r.	“In more than half of the samples of both genders, Li levels were below the instrumental detection limit or below or the lower limit of the laboratory reference. Li deficiency must be considered as potential suicide risk factors”
Helbich et al. (35)	Del Matto et al. (9)	Austria, 99 districts	All suicides 2005–2009 (total population 8 297 964)	11.3 μg/L (3.3–82.3 μg/L) [0.0016 mmol/L (0.005–0.0119 mmol/L)]	*n* = 6460; 2005–2010, from local drinking water; ICP-OES	T: 16.5 M: 26.4 F: 7.00	T: 0.790 M: 0.821 F: 0.673	Less suicide with higher [Li], only at lower altitudes Spearman's correlation of SMR on Li T: r = −0.26; *p* = 0.009 Moderating spatially filtered model - Intercept: 0.615; s.e. 0.125; t 4.908; *p* 0.000 - Li level:−9.407; s.e. 2.218; t−4.242; *p* 0.000 - Altitude:−0.000; s.e. 0.000; t−2.979; *p* 0.004 - Spatial filter: 1.030; s.e. 0.156; t 6.619; *p* 0.000 - Li-altitude interaction: 0.017; s.e. 0.008; t 2.271; *p* 0.026 - Akaike information criterion−111 - Adjusted R^2^ 0.554 (Spatial Filter 40%) - F-test: 21.30; *p* < 0.001 Covariates: Population density, income per capita, proportion of Roman Catholics, unemployment rates, density of GPs, psychotherapists and psychiatrist
Bluml et al. (36)	Del Matto et al. (9) and Memon et al. (10)	Texas, USA, 226 counties	All suicides 1999–2007 (total population n.r.)	46.3 μg/L (2.8–219.0 μg/L) [0.0067 mmol/L (0.0004–0.0316 mmol/L)]	*n* = 3123; 1999–2007, from public wells; analysis method n.r.	13.16	n.r.	Less suicide with higher [Li] PW linear regression of age-standardized suicide rate on log Li T: β = −0.04, s.e. = 0.02, *p* < 0.01 RR for fractional polynomial model: 0.88 for 100 μg/l; 95 %CI: 0.84, 0.93 Covariates: Population density, age, proportion of females, African Americans, Hispanics and Latino Americans, median income per household, poverty, unemployment
Giotakos et al. (37)	Barjasteh-Askari et al. (8), Del Matto et al. (9) and Memon et al. (10)	Greece, 34 prefectures	All suicides 1999–2010 (total population n.r.)	11.10 μg/L (0.1–121 μg/L) [0.0016 mmol/L (0.00001–0.0175 mmol/L)]	*n* = 149; during 2012; from drinking water from rural and urban areas; mass spectrometry	n.r.	n.r	Less suicide with higher [Li] Linear regression of age-standardized suicide rate on Li T: β= −0.02; t = −2.10; *p* < 0.05 Covarietes: n.r.
Sugawara et al. (38)	Barjasteh-Askari et al. (8), Del Matto et al. (9) and Memon et al. (10)	Aomori, Japan, 40 municipalities	All suicides 2008–2010 (total population 1 373 339)	n.r. (0–12.9 μg/L) [n.r. (0–0.0019 mmol/L)]	*n* = n.r.; date n.r., from tap water supplies; mass spectrometry	n.r.	M: 123 (96–186) F: 105 (72–152)	NA between [Li] and SMR PWLS regression of SMR on log Li Unadjusted analyses M: β = 0.136; *p* = 0.408 F: β = −0.350; *p* = < 0.05 Adjusted analyses M: β = 0.064; *p* = 0.777 F: β = −0.369; *p* < 0.10 Covariates: Density of medical institutions, unemployment rate
Helbich et al. (39)	Barjasteh-Askari et al. (8) and Del Matto et al. (9)	Austria, 99 districts	Alll suicides 2005–2009 (total population n.r.)	10 μg/L (3–27 μg/L) [0.0014 mmol/L (0.0004–0.0039 mmol/L)]	*n* = 6,460; during 2005-2010; from water samples; analysis method n.r.	15.7	79	Less suicide with higher [Li], in total population and males, but not in females Spearman's correlation of SMR on logLi T: r = −0.37; *p* < 0.001 M: r = −0.32; *p* = 0.003 F: r = −0.28; *p* = 0.009 Covariates: proportion of Roman Catholics; population density; average income per capita; density of psychiatrists; number of general practitioners; density of psychotherapists; average unemployment rates
Ishii et al. (40)	Barjasteh-Askari et al. (8) and Del Matto et al. (9)	Kyushu Island, Japan, 274 municipalities	All suicides 2011 (total population 14 646 121)	4.2 μg/L (0–130 μg/L) [0.0006 mmol/L (0–0.0188 mmol/L)]	*n* = 434; 2010–2013, from tap water samples (mainly from the main rail station or the municipal office); mass spectroscopy	T: 23.8 M: 35.3 F: 13.4	T: 114 M: 120 F: 101	Less suicide with higher [Li], in males but not in total population nor females PWLS regression of SMR on log Li Unadjusted analyses T: β = −0.175; *p* = 0.031 M: β = −0.228; *p* = 0.005 F: β = −0.004; *p* = 0.957 Adjusted analyses (model 1) T: β = −0.150; p = 0,041 M: β = −0.198; *p* = 0.007 F: β = −0.021; *p* = 0.795 Adjusted analyses (model 2) T: β = −0.122; *p* = 0.094 M: β = −0.169; p = 0.019 F: β = 0.031; *p* = 0.706 Covariates: Model 1: proportion of elderly people, proportion of 1-person households, proportion of people with college education or more and proportion of people engaging in primary industry. Model 2: all above and overall unemployment rate, annual marriage rate, annual mean temperature, and annual postal savings per person
Pompili et al. (41)	Barjasteh-Askari et al. (8), Del Matto et al. (9) and Memon et al. (10)	Italy, 145 cities	All suicides in ages >15, 1980–2011, except 2004–2005 (total population 17 200 000 in 2000–2011)	5.28 μg/L (0.11–60.8 μg/L) [0.0008 mmol/L (0.00002–0.0088 mmol/L)]	*n* = 157; 2009–2010; from samples of drinking water in public distribution systems; mass spectrometry by third party as part of a separate research	2000–2011: 7.53	n.r.	Less suicide with higher [Li] only in females from 1980–1989 PWLS regression of SMR on log Li 2000–2011: Unadjusted analyses T: β < 0.001; *p* = 0.997; M: β = 0.046; *p* = 0.581 F: β = −0.134; *p* = 0.109 Adjusted analyses T: β = 0.079; *p* = 0.308 M: β = 0.107; *p* = 0.159 F: β = −0.032; *p* = 0.703 1990–1999: Unadjusted analyses T: β = −0.047; *p* = 0.578 M: β = −0.009; *p* = 0.915 F: β = −0.165; *p* = 0.047 Adjusted analyses T: β = 0.079; *p* = 0.323 M: β = 0.087; *p* = 0.280 F: β < 0.001; *p* = 0.998 1980–1989: Unadjusted analyses T: β = −0.234; *p* = 0.005; M: β = −0.161; *p* = 0.053 F: β = −0.339; *p* < 0.001 Adjusted analyses T: β = −0.044; *p* = 0.560; M: β = 0.013; *p* = 0.859; F: β = −0.154; *p* = 0.043 Covariates: Mountainous, urbanized, south of Rome
Knudsen et al. (42)	Barjasteh-Askari et al. (8) and Del Matto et al. (9)	Denmark	All suicides in ages ≥ 21, 1991–2012 (total adult population 3 740 113). Data obtained from the nationwide individual- level Danish registers	11.6 μg/L (0.6–30.7 μg/L) [0.0017 mmol/L (0.0001–0.0044 mmol/L)] Exposure was calculated as a moving five-year TWA lithium exposure level for all individuals Group A: n.r. (2.0–7.0 μg/L) [n.r. (0.0003–0.0010 mmol/L)] Group B: n.r. (7.1–11.0 μg/L) [n.r. (0.0010–0.0016 mmol/L)] Group C: n.r. (11.1–15.0 μg/L) [n.r. (0.0016–0.0022 mmol/L)] Group D: n.r. (15.1–19.0 μg/L) [n.r. (0.0022–0.0027 mmol/L)] Group E: n.r. (19.1–27.1 μg/L) [n.r. (0.0028-0.0039 μg/L)]	n = 158; October 2009 to June 2010, and April to June 2013 from public waterworks; mass spectrometry	29.7 - 18.4	n.r.	NA, but SR decreased from 29.7 per 100,000 person-years in 1991 to 18.4 per 100 000 person-years in 2012 Poisson regression model with the random effect modeled using a conditional autoregressive model of IRR for suicide with increasing five-year TWA lithium exposure level Adjusted analyses T: Group A: IR = 19.9, IRR = 0.93 (CI95% 0.86–1.01) Group B: IR = 19.3, IRR = 0.91 (CI95% 0.83–0.99) Group C: IR = 21.0, IRR = 0.96 (CI95% 0.88–1.04) Group D: IR = 24.1, IRR = 1.00 (CI95% 0.92. 1.09) Group E: IR = 22.3, IRR = 1 (ref) Covariates: gender, ethnicity, age, employment, civil status, and calendar year.
Shiotsuki et al. (43)	Barjasteh-Askari et al. (8), Del Matto et al. (9) and Memon et al. (10)	Japan, Hokkaido Island and Kyushu Island, 153 cities	All suicides from cities only, 2010 – 2011 (total population 16 981 717)	3.8 μg/L (0.1–43 μg/L) [0.0044 mmol/L (0.00001–0.0062 mmol/L)]	*n* = n.r.; 2010–2015, from tap water samples (mainly from rail stations and city offices); mass spectrometry	T: 23.8 M: 35.7 F:13.1	T: 111.2 (26.9–268.8) M: 119.1 (0–245.0) F: 97.1 (0–319.0)	Less suicide with higher [Li] in males but not in total population nor females PWLS regression of SMR on log Li Unadjusted analyses T: β = −0.153; *p* = 0.059; M: β = −0.225; *p* = 0.005; F: β = −0.012; *p* = 0.883 Adjusted analyses T: β = −0.129; *p* = 0.070; M: β = −0.164; *p* = 0.037; F: β = 0.014; *p* = 0.870 Covariates: Annual mean temperature, total sunshine, total rainfall and total snowfall
Liaugaudaite et al. (44)	Barjasteh-Askari et al. (8), Del Matto et al. (9) and Memon et al. (10)	Lithuania, 9 cities	All suicides 2009–2013 (total population 1 109 261)	10.9 μg/L (0.48 - 35.53 μg/L) [0.0016 mmol/L (0.0001–0.0051 mmol/L)]	*n* = 22; Nov 2013 to Jan 2014, from public water supply system; mass spectrometry	n.r.	T: 27 (range 16–50) M: 51 (range 29– 93) F: 7 (range 0–13)	Less suicide with higher [Li], in total population and males, but not females PWLS regression of log Li on age- standardized suicide rate Unadjusted analyses T: β = −0.911; *p* = 0.156 M: β = −0.965; *p* = 0.100 F: β = 0.150; *p* = 0.374 Adjusted analyses T: β = −0.283; *p* = 0.034; M: β = −0.702; *p* = 0.013; F: β = 0.253; *p* = 0.523 Covariates: Female:male ratio of city population
Fajardo et al. (45)	Del Matto et al. (9)	Texas, USA, 254 counties	All cause mortality (253 counties) and all suicides (140 counties) 2006–2015, and all premature deaths 2011–2016 (214 counties)	n.r. (3–539 μg/L) [n.r. (0.0004–0.0778 mmol/L)]	*n* = 6,180; since 2007 from water samples from public wells; method n.r.	n.r.	n.r.	[Li] negatively associated with all causes mortality, and years of potential life lost Spearman's correlation of age-adjusted all cause mortality on log Li Unadjusted analyses T r = −0.18; *p* = 0.006 Adjusted analyses (model 1) T: r = −0.11; *p* = 0.19 Adjusted analyses (model 2) T: r = −0.26; *p* = < 0.0001 Pearson's correlation of years of potential life lost on log Li Unadjusted analyses T:: r = 0.22; *p* = 0.001 Adjusted analyses (model 1) T: r = −0.22; *p* = 0.01 Adjusted analyses (model 2) T: r = −0.17; *p* = 0.009 Covariates: Model 1: suicide mortality. Model 2: suicide mortality, and median household income, unemployment rate, adults with college education rate
Oliveira et al. (46)	Barjasteh-Askari et al. (8) and Del Matto et al. (9)	Portugal, 54 municipalities	All suicides 2011–2016 (total population n.r..)	10.88 μg/L (0–191 μg/L) [0.0016 mmol/L (0–0.0275 mmol/L)]	*n* = 54; 2011–2014, from public drinking water samples; mass spectrometry	7.5	119 (0–712)	NA between [Li] and SMR Pearson correlation between Li and SMR Adjusted analyses T: r = 0.001; *p* = 0.996 M: r = 0.024; *p* = 0.862 F: r = 0.000; *p* = 0.999 PWLS regression of logLi and SMR Adjusted analyses NA (data n.r.) Covariates: population density, average income per capita, unemployment rates and proportion of Roman Catholics
Palmer et al. (47)	Barjasteh-Askari et al. (8), and Memon et al. (10)	Alabama, USA, 15 counties	Average suicide rate 1999–2013 (total population n.r.)	n.r. (0.4–32.9 μg/L) [n.r. (0.0001–0.0047 mmol/L)]	*n* = 75; May 2016 from public locations; plasma emission spectrophotometry	1.28 (3.3–22.0)	n.r.	Less suicide with higher [Li] in total population and males, but not in females Spearman's correlation of age-standardized suicide rate against Li levels T; r = −0.6286; *p* = 0.0141; M: r = −0.625; *p* = 0.0148; F: r = −0.4393; *p* = 0.1032 Linear regression of SMR on log Li Unadjusted analyses T: β = −0.6188; s.e. = 0.2179 M: β = −0.6236; s.e. = 0.2168 F: β = −0.4387; s.e. = 0.242 Covariates: age, gender, and the percent of each county's population below established poverty rate

*Characteristics of the ecological studies included in the reviews and meta-analysis available of lithium at trace doses in drinking water as a suicide protective agent (8–10). The information presented is adapted directly from Barjasteh-Askari et al. (8), Del Matto et al. (9) and Memon et al. (10), and complemented from each primary study*.

*SR, suicide rate; SMR, standardized mortality ratio; n.r., not reported; NA, no association; Li, lithium; [Li], litihum concentration; T, total (both genders combined); M, male; F, female; IR, incidence rate; PWLS, population-weighted least squares; ICP-OES, inductively coupled plasma optical emission spectrometry; GP, general practitioner; s.e., standard error; CI, confidence intervals; RR, rate ratio; IRR, adjusted incidence rate ratio; TWA, time-weighted average; ref, reference group*.

Using a similar approach as Knudsen et al. (42), Kessing et al. reported that the incidence rate ratio of mania/bipolar disorder did not decrease with higher long-term lithium exposure (48) but may be associated with a lower incidence of dementia (49).

Few studies have reported adverse effects of lithium in trace doses. In a clinical sample from an Argentine community in the Andes Mountains, whose levels of lithium in drinking water were around 1,005 μg/L (0.1448 mmol/L), Broberg et al. (50) reported no association between lithium and thyroxine or thyroid stimulating hormone values outside the normal range. Harari et al. (51) in the same community, found an inverse association between lithium levels in maternal blood and urine, and fetal measurements. An increase of 100 μg/L (0.0144 mmol/L) of lithium in maternal blood was associated with neonates measuring approximately 2 cm smaller.

## Water Fluoridation: Experiences From a Similar Public Policy

There are established and successful examples of water supplementation, such as fluoridation. This measure was implemented in 1945 in the United States for the prevention of dental caries, according to the benefits found in the 1930s and 1940s (52). Dean et al. (53, 54) showed that dental caries decreased when the level of natural fluoride increased from low to normal levels (<0.1 mg/L). Based on this and other studies, the first community-controlled water fluoridation trial was conducted in Grand Rapids, with the nearby city of Muskegon acting as a control (52).

The first dental data from the Grand Rapids-Muskegon study was published in 1950, based on information collected in 1944-45 based on a population of 28,614 children in Grand Rapids and the 7,786 children in Muskl Kegon aged 4 years. Finally, 15 years after the total experience, it was concluded that total caries was reduced by 50–63% in children between 12 and 14 years old, and by 48–50% in children between 15 and 16 years old in comparison to the control region (55).

Based on these data, in 1950, the Director of Dentistry of the United States Public Health Service issued a statement to the American Dental Association endorsing water fluoridation as a public policy subject to state and local health authorities (52). For its part, in 1958 the UN expert committee concluded that "drinking water containing approximately 1 ppm of fluoride (1 mg/L) has a marked caries preventive action (...)” (56). By 1960, the water fluoridation policy was widely implemented despite the questions arising from the initial study (52). Currently, the safety and effectiveness of water fluoridation have been internationally supported by technical organizations such as the World Health Organization (WHO), the National Research Council US, among others (57). In the case of WHO, the entity has established certain technical prerequisites for the correct implementation of this measure, including an optimum level of 1 ppm (58, 59). Currently, the debate has also arisen around the possible long-term adverse effects (59) as well as the ethical dilemmas that it entails, such as the passive role of the communities throughout the implementation process, which was previously considered an advantage (57, 59). Among the countries where this public policy has been implemented, it has been reported that some groundwaters contain particularly high concentrations of fluoride above the optimum level (58, 59). At the present time, the fluoridation of water is a practice carried out in about 25 countries around the world (52, 58, 59).

## Discussion

The supplementation of drinking water with lithium as a public policy for suicide prevention is a controversial issue that involves several considerations and will necessarily produce debate. According to the Nuffield Council on Bioethics (60), any measure that involves water supplies must consider three elements: (I) The balance of risks and benefits; (II) The possible alternatives that require lower range of intervention to achieve the same goal (III), and the role of consent in case of possible damages. These are summarized in Table 2.

**Table 2:** What makes water supplementation acceptable as a public policy?

	**Aspects in favor of adding lithium in drinking water**	**Aspects against adding lithium in drinking water**
**1.- Balance of risks and benefits**		
Lithium in drinking water could generate public health benefits	There is an inverse association between trace dose levels of lithium in drinking water and the suicide mortality rate in women and the total population (8–10).	There is susceptibility to ecological fallacy in the meta-analysis of ecological studies. Memon et al. propose conducting randomized clinical trials supplementing water supplies with lithium (10).
Efficacy of low-dose lithium at the population level appears to be better than a selective intervention	Individual risk of committing suicide is difficult to know in advance. Many times, health systems and services do not have enough tools for timely and effective help (7). Population-level interventions to prevent suicide are highly cost-effective in most scenarios (61).	Suicide is relatively a rare event, where supplementation of drinking water with lithium would act in a mostly low-risk population.
Trace amounts of lithium appear to have little or no serious adverse effects	Adverse effects from lithium are dose dependent (12). Serious renal adverse effects are rare and associated with chronic use for decades (22).	Broberg et al. (50) reported an association between urinary lithium concentration and thyroid function markers. However, no association was found between lithium and thyroxine or thyroid stimulating hormone values outside the normal range. Harari et al. (51) reported an association between lithium levels in maternal blood and urine, and lower fetal measurements.
The practice of fortifying food and drinking water is already established and successful.	There are examples such as water fluoridation, food supplementation with vitamins and minerals, at an international level (57, 59, 60)	
Lithium's adherence to drinking water is not that different in principle from current fortification practices		
**2.- The potential of alternatives that are of a lower range of intervention to achieve the same goals**		
Alternative 1: Table salt (62).	Risk of stigmatization: choosing salt rich in lithium can lead to stigmatization of the population that consumes it. Ignorance can discourage the purchase of fortified foods. The benefits would be less compared to a more massive policy like water supplementation	Table salt would involve lower risk of exposure to potentially vulnerable populations (for example, children)
Alternative 2: General medication (62).	General medication leaves unprotected people at risk who do not have access to health services, especially mental health.	In high-risk patients, prophylactic measures such as lithium as general medication are taken in those with suicidal considerations or with high-risk factors. As it is a choice, it is possible to obtain consent. Adverse effects end up being more justified since they are high-risk patients, and it allows monitoring.
Alternative 3: Individual prescript (62)	The population impact would be quite low with individual prescription. Evidence regarding the dose associated with reducing suicide risk is still heterogeneous and limited (9, 11).	Similar to general medication, individual prescription could be offered to patients at high risk of suicide, with the possibility of obtaining consent, and with adverse effects being more justified than a general measure.
**3.- The role of consent in the event of possible harm**		
Confidence level in the measure to be implemented		Distrust can alter the implementation of population-based health measures, especially where there is a lack of credibility in the authority (60, 62), and the substance to be added is psychotropic, such as lithium.
Collateral consequences		Collateral consequences should be considered, such as increased trade in lithium-free water leading to more monetary expenses, environmental waste, or increased consumption of sugary beverages due to avoiding drinking water (62).

*The three elements to consider that make any measure involving water supplies acceptable are presented, according to the Nuffield Council on Bioethics Hepple and Ng et al. (60, 62)*.

Applying the balance of risk and benefits prepared by Ng et al. (62), the argument for supplementing drinking water with lithium could be understood from five points. First, population-level trace doses of lithium could generate public health and economic benefits by reducing suicide rates (8–10) and its externalities. Moreover, it could also have a role as a mood stabilizer (with limited evidence only in patients with history of substance use disorder) (19) and as a neuroprotector (14, 49), potentially saving some of the cost that mood and cognitive disorders have for society and health systems. Second, individual risk of committing suicide is difficult to know in advance, and many times health systems and services do not provide enough tools for timely and effective help (7), the efficacy of low-dose lithium at the population level appears to be better than a selective intervention. Third, trace amounts of lithium appear to have insignificant adverse effects (50, 54), however, further research on its effects should be continued on both individual and a collective level. Fourth, the practice of fortifying drinking water is well established and successful (57, 59). Fifth, lithium's addition to drinking water is in principle not that different from current fortification practices.

Measures such as supplementation of the table salt, general medication, or individual prescription may appear as possible alternatives that require less range of intervention (62). For example, while the entire population is susceptible to cavities, therefore fluoridation is a measure applicable to the entire population (57, 59), supplementation of drinking water with lithium acts in a mostly low-risk population. However, it must be considered the effectivity cost of targeted interventions that require finding high-risk populations and offering them individualized treatment to achieve the same goals that a population-level intervention may achieve (61). It also should be considered the potential mood stabilizer (19, 20) and neuroprotective effect (14, 49) that trace doses of lithium may exert, being mood and cognitive disorders highly prevalent in the general population (63, 64).

When addressing the role of consent in case of possible damages, the Nuffield Council on Bioethics (60) states that the events that are sought to be prevented (suicides) are not the result of autonomy or rational decision-making, especially in people with psychiatric disorders. Distrust toward the measure to be implemented and collateral consequences are some of the practical challenges that must be considered. On one hand, mistrust can alter the implementation of population-based health measures, especially where there is a lack of credibility in the authority (60, 62), and the substance to be added is a psychotropic such as lithium. On the other hand, the collateral consequences that a measure like this can produce must be considered. Even though the studies available (50, 51) suggest that a policy like this may have insignificant adverse effects, there is still a lack of knowledge about its overall long-term effect. In these cases, the most appropriate way to decide on water supplementation would be through democratic decision-making procedures (60), where ideally these are in accordance with the need and perception of lithium supplementation in different locations and not a national measure. If so, responsible state agencies should monitor the effects of gradually introduced water supplementation (60), including the incidence and severity of excess lithium and other possible harm.

Water supplementation shows tensions between competing principles. The Nuffield Council on Bioethics consider mainly three principles in favor of supplementation (60): (I) Reducing the risks of becoming ill: States must provide interventions that improve the health of their population. The supplementation of water, in this case, would improve environmental conditions, promoting greater health of the population. This type of argument has made it possible to justify the fluoridation of water (57, 59), in addition to adding compounds that filter harmful substances (60); (II) Protect the health of the most vulnerable: One of the most vulnerable groups to die by suicide corresponds to those with mental disorders (1, 5). However, it must be considered that even though one can justify the vulnerability of these groups, it is not appropriate for the State to promote health in such a way that it infringes the freedoms of third parties. Water supplementation may be a special case in which vulnerable groups could be reached directly without major infringements on other's liberties (60) such other measures as restricting access to pesticides and firearms, or designing policies aimed at reducing alcohol consumption (1); (III) Reduce health inequities: Socioeconomic levels are among the factors that influence suicide rates at a population level (4). Reducing inequities in health should be considered a central goal in public health. A measure such as the supplementation of water would be transversal to the different social groups (60).

The Nuffield Council on Bioethics address four principles against supplementation (60): (I) Damage prevention: As mentioned before, few studies have reported adverse effects of lithium in trace doses (57, 59), with an overall lack of knowledge about its long-term effect. Ng et al. propose that harm-based objection is based on four variants (62): (a) Aggregate individual effects: Adverse effects at the individual level increase and accumulate as more people are exposed. Despite this, it must be considered prioritize prevention of large harms, such as suicide, over prevention of much smaller ones regardless of aggregative benefits; (b) Collective effects: Some may theorize that there is risk of dismissing the causes that lead to the problem of suicide. Nevertheless, suicide is multifactorial phenomenon (1, 2), in which focalized interventions should not be undermined; (c) Active nature: Harm from naturally occurring lithium might be considered less weighty than ones introduced by the State. However, one can discuss if the harm actively introduced outweights the harm that is being prevented; (d) Distribution effect: Adding lithium would not increase the benefit and would put an increased risk for some groups. For example, the child population (51), with little risk of suicide, will be exposed at an early age to this measure. Despite this, the vast majority of the population is exposed to the externalities of suicide (1, 61), such as the collateral effects on the mental health of relatives, the consequences for health systems, and the economic losses. In this context, water supplementation should be gradually implemented with and strict monitoring of any adverse effect in the short and long-term. (II) Do not intervene without the consent of those affected: Obtaining consent is important in medical interventions. However, it is not possible to accommodate this implementation to every individual in the area (57). The discussion should be focused on the requirements of how to obtain general consent through procedures that can reconcile the preferences of the population (59, 60). (III) Minimize interventions that affect important areas of life: There is the conception of water as “pure” or “natural” and, therefore, it should not be intervened. Nevertheless, its composition already varies from place to place, with treatment and sanitation processes involved. Public opinion has a good acceptance of the addition of products that are beneficial (60). (IV) Do not coerce adults to lead healthy lives: while it should not normally be considered acceptable to restrict freedoms to force individuals into leading healthy lives, it should be considered the amount of freedom to be sacrificed, with individuals taking a passive role with a measure like water supplementation (60).

Regarding what lithium concentration in drinking water should be aimed at, the data described in the ecological studies is highly heterogeneous and limited, with ranges from 0.1 to 539 μg/L (0.00001 to 0.0777 mmol/L), with studies including only high-income countries. As mentioned before, Knudsen et al. (42), in their analysis of lithium exposure on an individual level calculated as a moving five-year time-weighted average, suggested that there is not seem to be a protective effect below 30.7 μg/L (0.0044 mmol/L). This can be considered consistent with the other ecological studies, which report a significant association of lower risk of suicide with higher lithium levels, up to 32.9 μg/L (0.0047 mmol/L) to 219 μg/L (0.0316 mmol/L). In the same way, ecological studies with lower levels of lithium up to 12.9 μg/L (0.0019 mmol/L) to 21 μg/L (0.0030 mmol/L) did not find a significant association. This information should be cautiously pondered, considering that suicide is a complex phenomenon in which lithium levels in drinking water needed to reduce suicide may differ in each locality, with no clear evidence of blood lithium levels needed to be reached. In this context, making cost analysis for the application of such a public policy requires a multifactorial analysis of the aspects previously mentioned, starting with a consensus on lithium concentration in water or at least, an “optimum level,” as happened with water fluoridation (32), where the suicide rates start to reduce.

In the same way as the fluoridation process, this measure requires endorsing state and local health authorities for the development of community-controlled trials, while establishing the necessary technical prerequisites for the correct implementation and strict monitoring of any adverse effect. In this context, the amount of lithium needed to be gradually introduced should be aimed according to the reality of each community based on their local context, and should be explored in future research.

## Conclusions

Suicide is a multifactorial and major public health problem. The supplementation of drinking water with lithium for its prevention is a controversial issue that will necessarily produce debate.

This paper was aimed as a proposal to discuss the addition of lithium to drinking water to decrease the suicide rate in order to open a discussion that seems necessary and pertinent. Having had successful experiences of similar public policies in water supplementation and the current evidence available, we believe it is extremely important to continue researching in this area.

Future challenges may involve establishing a consensus on lithium concentration in water or at least, an “optimum level,” and developing community-controlled trials with strict monitoring of any adverse effect, while democratic decision-making procedures at the level of the different localities would constitute one of the most appropriate ways to validate the implementation of a measure like this, according to their needs and perceptions of each community. When discussing this, we must not forget to present the information in a balanced way, considering risks and benefits, as well as making the certainty of the available evidence transparent, which will allow us to evaluate a policy like this critically and responsibly.

## Data Availability Statement

The original contributions presented in the study are included in the article/supplementary material, further inquiries can be directed to the corresponding author.

## Author Contributions

PA, CM, and JB: article concept and design, literature research, drafting of the manuscript, critical revision, and final approval of the manuscript. All authors contributed to the article and approved the submitted version.

## Conflict of Interest

The authors declare that the research was conducted in the absence of any commercial or financial relationships that could be construed as a potential conflict of interest.

## Publisher's Note

All claims expressed in this article are solely those of the authors and do not necessarily represent those of their affiliated organizations, or those of the publisher, the editors and the reviewers. Any product that may be evaluated in this article, or claim that may be made by its manufacturer, is not guaranteed or endorsed by the publisher.
